# Assessment of Nutritional Risk at ICU Admission and Mortality in Patients with Suspected COVID-19

**DOI:** 10.3390/clinpract12060100

**Published:** 2022-11-23

**Authors:** Gustavo D. Pimentel, Claude Pichard, Paula M. Martins, Emanoelly P. Franco

**Affiliations:** 1 Faculty of Nutrition, Federal University of Goias, Rua 227, Quadra 68 s/n°, Setor Leste Universitário, MA 74605080, Goiânia 74605-080, Brazil; 2 Clinical Nutrition, Geneva University Hospital, 1211 Geneva, Switzerland; 3 Clinical Hospital, Federal University of Goias, Goiânia 74605-050, Brazil

**Keywords:** COVID-19, malnutrition, nutritional status, ICU, mortality

## Abstract

Background/Objectives: The association between the nutritional risk and mortality in Brazilians with COVID-19 is poorly documented. Therefore, this study, for the first time, aimed at investigating the length of stay in the ICU and the chance of dying in patients with suspected COVID-19, without and with nutritional risk. Subjects/Methods: This retrospective monocentric study enrolled adult, COVID-19-positive patients that were admitted to the ICU at a university hospital. Biochemical analysis and clinical data were collected from medical records and the nutritional risk was assessed according to the Modified-Nutrition Risk in the Critically Ill (mNUTRIC) score. The Cox model was used to assess the chance of mortality in the patients with and without nutritional risk. Results: Out of 71 patients, 63.3% were male and 52% were older (≥60 years). Although no differences were found between groups for the length of stay in ICU, C-reactive protein, alanine aminotransferase and aspartate aminotransferase concentrations, the mNUTRIC ≥ 5 group had higher D-dimer than the mNUTRIC < 5 group. Regarding ICU mortality, most patients (69.5%) in the mNUTRI ≥ 5 group died while in the mNUTRIC < 5 group 33.3% died (*p* = 0.0001). In addition, patients with mNUTRIC ≥ 5 had (HR: 2.04 [95% CI: 1.02–4.09], *p* = 0.04) a more likely chance of dying than patients in the mNUTRIC < 5 group, even that adjusted by BMI and D-dimer concentrations (HR: 2.18 [95% CI: 1.04–4.56], *p* = 0.03). Conclusion: In patients with COVID-19, an mNUTRIC ≥ 5 score at admission leads to a more likely chance of death even after controlling for confounding variables.

## 1. Introduction

Lew and co-authors [1] found in a systematic review a prevalence of 38–78% of malnutrition in patients admitted to intensive care units (ICU). In addition, the authors observed that malnutrition is a risk factor independently associated with the worst clinical outcomes during the ICU stay [1].

Three previous studies tested the use of the modified-NUTRIC (mNUTRIC) by excluding the IL-6 values in the evaluation of nutritional status in patients with COVID-19 and/or critically ill [2,3,4]. In addition, a recent study showed that the Nutrition Risk score 2002 (NRS) and mNUTRIC scores are useful to assess the nutritional risk in patients with COVID-19 since their values are associated with poor clinical outcomes during the ICU stay [4]. The nutritional risk due to anorexia and wasting are considered causes of death in the ICU. However, the evaluation of biochemical analysis, length of stay in the ICU and the association between nutritional risk and mortality in Brazilians with COVID-19 is fully limited.

Thus, this retrospective monocentric study, for the first time, aimed to investigate the length of stay in the ICU and the chance of dying among Brazilian patients with suspected COVID-19, without and with nutritional risk.

## 2. Materials and Methods

### 2.1. Design of Study

**Figure 1:**
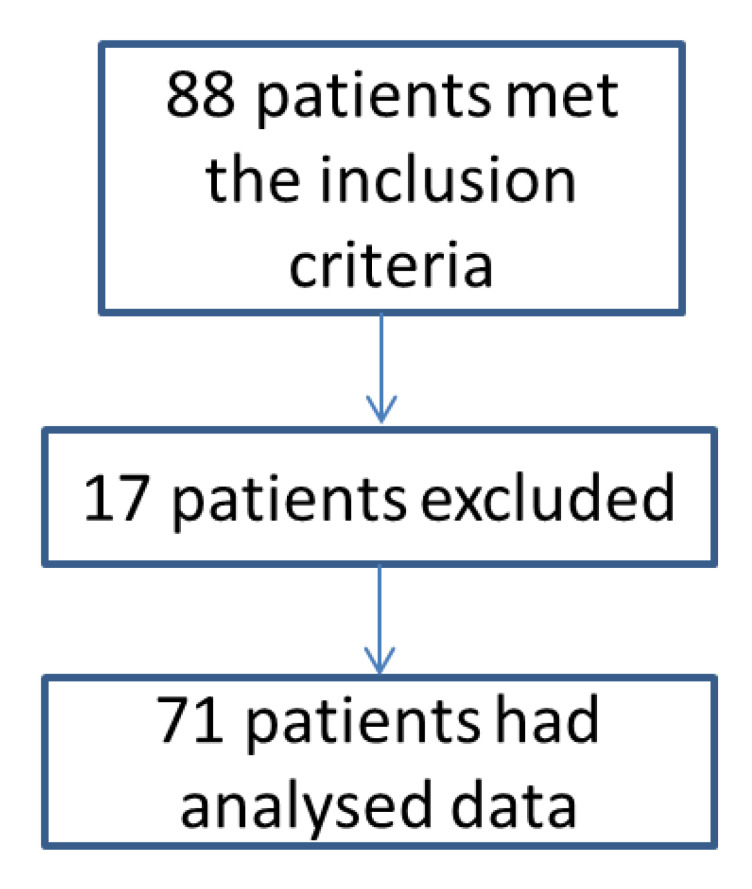
Flowchart study.

A retrospective monocentric study enrolled adult and elderly suspected COVID-19 patients that were admitted to the ICU at a university hospital. This protocol was approved by the Research Ethics Committee of the Clinical Hospital under the number 4.381.491. The inclusion criterion was patients with COVID-19 RT-PCR positive and the exclusion criteria were patients without available data to calculate the mNUTRIC score. The study was conducted from March 2020 to October 2020. Out of 88 patients who met the inclusion criteria, 17 were excluded because of incomplete data from the mNUTRIC tool (Figure 1).

### 2.2. Data Collection

Data were collected from medical records during the first 48 h of hospitalization at the ICU regarding sex, age, body weight, height, length of stay in ICU days, mortality, modified nutritional risk questionnaire (mNUTRIC) [5] and biochemical data (CRP, D-dimer, alanine and aspartate aminotransferase). CRP concentrations were quantified by the biochemical immunoturbidimetric method. Hepatic enzymes were analyzed by ultraviolet kinetics. Body Mass Index (BMI) and mNUTRIC were measured to assess nutritional status. mNUTRIC by the exclusion of IL-6 values was developed to identify nutritional risk patients that consisted of a combination of APACHE, SOFA scores, age, number of comorbidities and pre-ICU length of hospitalization; thus, mNUTRIC may be used to predict mortality in critically ill patients, whereas mNUTRIC ≥ 5 score predict a high nutritional risk [4,5].

### 2.3. Statistical Analyses

The normality test was done using the Shapiro–Wilk test. Distribution variables were presented as mean and standard deviation or as median, minimum and maximum values. Normal distribution variables were tested using the Student’s *t* test and non-normal distribution variables were tested using the Mann–Whitney U test. Cox proportional-hazard regression (Model crude and Model 1 adjusted by BMI and D-dimer) was used to investigate mortality according to the mNUTRIC score. All analyses were done using the Medcalc^®^ software (Ostend, Belgium, version 11.1.1.0) and the significance level was set at 5%.

## 3. Results

Out of 71 patients, 63.3% were male and 52% were older than 60 years. There is no difference between groups for sex, length in ICU, mechanical ventilation duration, BMI, blood alanine aminotransferase, aspartate aminotransferase and CRP (Table 1). Although there was no difference among the groups in the presence of comorbidities, most patients were admitted to ICU with diabetes, obesity, hypertension and cardiovascular diseases. However, those with mNUTRIC ≥ 5 were older and had higher D-dimer compared with patients in the mNUTRIC < 5 group (Table 1). Additionally, the length of stay in the ICU was similar among the groups.

Regarding ICU mortality, most patients (69.5%) in the mNUTRI ≥ 5 group died, while in the mNUTRIC < 5 group, 33.3% died (*p* = 0.0001) (Table 1). In addition, patients with mNUTRIC ≥ 5 had (HR: 2.04 [95% CI: 1.02–4.09], *p* = 0.04) a more likely chance of dying than patients in the mNUTRIC < 5 group, even with adjusted BMI and D-dimer concentrations (HR: 2.18 [95% CI: 1.04–4.56], *p* = 0.03).

## 4. Discussion

In the present study, patients with a nutritional risk (mNUTRIC ≥ 5) were 2.1% more likely to die than patients with mNUTRIC < 5.

Although the Nutrition Risk in the Critically Ill (NUTRIC) score does not include nutritional variables and is not recommended by international guidelines, which endorses only the Nutrition Risk score (NRS) [6], the present work and previous evidence [2,3,4] suggests the use of mNUTRIC in clinical routine, mainly in developing countries, such as China [2,4]. However, the present study was conducted in an underdevelopment country which was suffering an unprecedented economic and health challenge.

With respect to inflammation markers, patients critically ill with NRS ≥ 3 had high CRP and IL-6 concentrations [7]. However, using the mNUTRIC tool we did not find any difference in blood CRP concentrations among the mNUTRIC ≥ 5 or <5 groups.

In agreement with our findings, a retrospective and observational study performed in Wuhan, China, found that in patients critically ill and with higher NRS scores, there was a higher risk of mortality [7]. In addition, a study conducted in China revealed that patients with COVID-19 and a high nutritional risk had an increased probability of death in the ICU in 28 days (HR: 2.0) compared to a low nutritional risk [2]. Additionally, Zhang, et al. [2] used the same NUTRIC tool to screen the nutritional risk and found that 61% of patients at ICU admission were classified with high nutritional risk. Similarly, we found that 42.2% of patients had a nutritional risk when evaluated by NUTRIC ≥ 5.

Indeed, our data support the onset target of ICU admission that nutritional status evaluation is required to start nutritional support [8]. In addition, malnutrition can be aggravated by muscle loss, mainly in patients with COVID-19 admitted to the ICU when suffering from mechanical ventilation, continuous positive airway pressure, and opioid use [9]. Likewise, the use of nutritional support in patients with COVID-19 is further to be investigated [9].

### 4.1. Limitations

This design of the study cannot report the relation of causality and the limited sample size does not allow generalization of these data for all patients infected with SARS-CoV-2, since we evaluated patients with suspected COVID-19 during the initial pandemic period (March–October 2020). A debate still is ongoing about the use of the NUTRIC score because it does not include nutritional variables and is not recommended by international guidelines [6].

### 4.2. Strengths

Our study highlights the value of testing the nutritional risk at ICU admission using the mNUTRIC score, a simple and validated tool for evaluating patients admitted to the ICU, since the mNUTRIC score is a useful tool during the clinical practice and its values has been associated with mechanical ventilation, clinical outcomes, length of hospitalization, inflammation and death [2].

## 5. Conclusions

In patients with suspected COVID-19, a mNUTRIC ≥ 5 score at admission was 2.1 more likely to die than patients with mNUTRIC < 5, even after adjusting for BMI and D-dimer. Therefore, our study supports the use of the mNUTRIC score within the first 48 h at the ICU admission of patients to predict mortality and plan early nutritional support.

## Figures and Tables

**Table 1:** Socioeconomic and clinical variables of patients with suspected COVID-19 admitted to the ICU according to NUTRIC classification.

Variables	NUTRIC < 5 (n = 41)	NUTRIC ≥ 5 (n = 30)	*p*
Sex, n (%)			0.80
Male	25 (61)	20 (66.7)	
Female	16 (39)	10 (33.3)	
Age (years) ‡	49.6 ± 16.1	65.3 ± 13.5	<0.0001
Comorbidities, n (%)			0.67
Diabetes	10	12	
Hypertension	15	20	
Obesity	16	10	
Cardiovascular diseases	9	16	
COPD	6	5	
Chronic kidney disease	4	6	
Cancer	4	5	
Liver cirrhosis/steatosis/hepatitis	1	4	
Others	5	8	
Length of stay in ICU (days) ‡	9.6 ± 7.7	12.0 ± 7.8	0.10
Mortality, n (%)			0.0001 *
Alive	29 (66.6)	6 (30.5)	
Died	12 (33.3)	24 (69.5)	
Mechanic ventilation duration (d) ‡	12.5 ± 7.5	13.2 ± 8.7	0.38
Body mass index (kg/m^2^) ‡	29.9 ± 6.3	27.5 ± 6.4	0.06
NUTRIC (score) ‡	2.3 ± 1.3	6.8 ± 1.4	<0.0001 *
Biochemical analysis			
Alanine aminotransferase (U/L) †	44 (11–738)	45 (15–1069)	0.29
Aspartate aminotransferase (U/L) †	42 (12–874)	45 (24–2049)	0.19
D-dimer (ng/mL) †	623.5 (140–4068)	850.5 (0.6–25799)	0.04 *
C reactive protein (mg/dL) ‡	14.4 ± 9.3	17.1 ± 12.3	0.14

APACHE: Acute Physiology and Chronic Health Disease Classification System, COPD: Chronic obstructive pulmonary disease, ICU: intensive care unit, NUTRIC: Nutrition Risk in the Critically Ill, SOFA: Sepsis-Related Organ Failure Assessment; Cardiovascular diseases: including the insufficiency cardiac, stroke, chagasic cardiomyopathy, acute myocardial infarction, arrhythmia and angina pectoris; Other comorbidities: including dementia, schizophrenia, hypothyroidism, asthma, pancreatitis, intestine failure, gastroesophageal reflux disease, muscle atrophy and depression. * *p* < 0.05 was considered as significant. ‡ data are expressed as means and standard deviation. † data are expressed as medians and p25 and p75th.

## Data Availability

Data is available upon request to the corresponding author.
